# Post-kala-azar dermal leishmaniasis in HIV-positive patients: A study of two cases

**DOI:** 10.4103/2589-0557.69001

**Published:** 2010

**Authors:** Sejal Shah, Aditya Shah, Sachin Prajapati, Freny Bilimoria

**Affiliations:** Department of Dermatology and Venerology, SBKS Medical College and Research Centre, Pipariya, Waghodia, Gujarat, India

**Keywords:** HIV, post-kala-azar dermal leishmaniasis, Leishman Donovan bodies

## Abstract

Cutaneous leishmaniasis and human immunodeficiency virus (HIV) co-infection is emerging as increasingly frequent and serious new disease. Leishmaniasis may be acquired before or after HIV infection. We describe two cases of post-kala-azar dermal leishmaniasis in HIV-positive patients. Both the patients had papulonodular lesions on upper extremities and back with low CD4 count. Slit skin smear with giemsa stain revealed Leishman Donovan (LD) bodies and skin biopsy of both the patients revealed lymphohistiocytic infiltrate with numerous intracytoplasmic LD bodies.

## INTRODUCTION

Leishmaniasis is a parasitic disorder transmitted by bite of infected female phlebotomus sand fly in developing countries.[1] In human beings, the disease present in four different forms with a broad range of clinical manifestation: visceral leishmaniasis, or kala-azar, cutaneous leishmaniasis, mucocutaneous leishmaniasis, and diffuse cutaneous leishmaniasis.[2]

Lieshmaniasis human immunodeficiency virus (HIV) co-infection is very common in leishmania endemic countries. In India, it is endemic in Bihar, West-Bengal, Orissa, and some parts of Rajasthan. Leishmaniasis emerges as third most common opportunistic infection.[2]

Post-kala-azar dermal leishmaniasis (PKDL) is mainly seen in Sudan and India where it follows treated visceral leishmaniasis in 50% and 5-10% of cases respectively. Thus, it is largely restricted to areas where leishmania donovani is the causative parasite.[3]

In PKDL, the rash develops after the visceral disease has healed either spontaneously or following the treatment. A small proportion of patient does not give history of visceral disease.

Skin lesions mainly involve cheeks, chin, ears, external aspect of hands and forearms, buttocks, and lower legs. Usually, there are papules, nodules, and hypopigmented macules.

We describe two cases of PKDL in HIV-positive patients.

## CASE REPORTS

### Case 1

**Figure 1:**
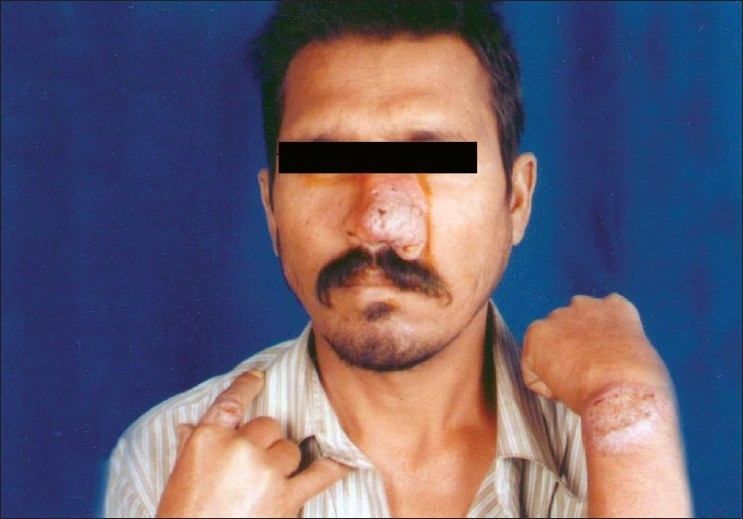
Infiltrated plaques on nose, left dorsum of wrist and on index finger of right hand

A 45-year-old HIV-positive man presented with nodules and plaques on nasal area, dorsum of left wrist, right index finger, and back with cervical lymph adenopathy [Figure 1]. Patient had history of fever 6 months back when he was working in state of Bihar. Patient also complained of mild pyrexia off and on and weight loss. Routine blood and urine examination did not reveal any abnormalities. Enzyme-linked immunosorbent assay test for HIV was positive, and CD4 Count was 210/*μ*L. Slit skin smear with giemsa stain revealed abundant intra- and extracellular leishman donovan bodies.

Skin biopsy was taken from lesions on back, which shows dermal infiltrate composed of lymphocytes and histiocytes with numerous intracytoplasmic leishman donovan bodies.

### Case 2

**Figure 2:**
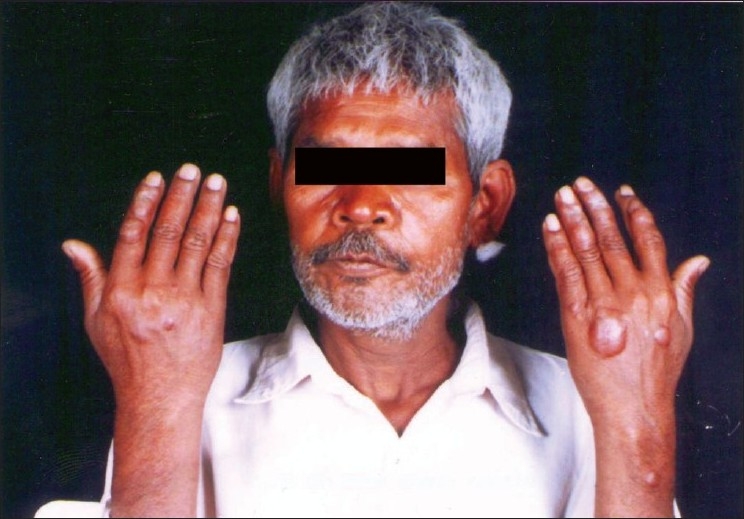
Nodular lesions on dorsum of both hands

**Figure 3:**
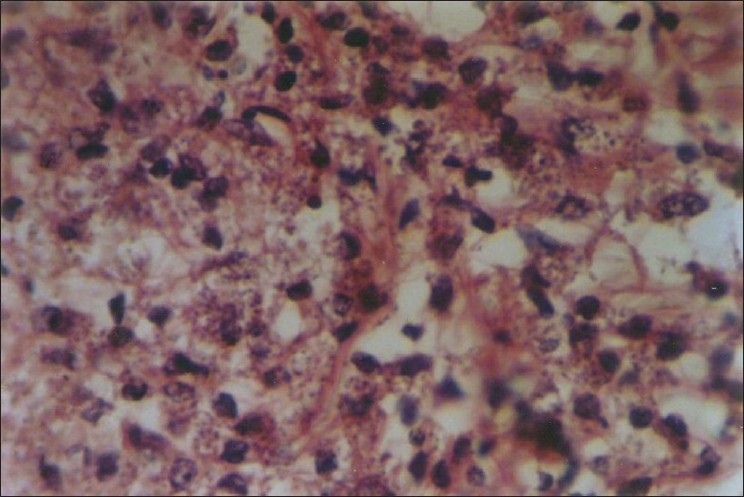
H and E shows macrophages containing leishman donovan bodies

A 52-year-old HIV-positive man from Rajasthan presented with multiple papulonodular lesions mainly on dorsum of hand, forearm, thighs, and back for past 3 months [Figure 2]. Differential diagnosis of Hansen’s disease, sarcoidosis, and leishmaniasis were performed. Routine blood parameters were in normal limits. His CD4 count was 170/*μ*L. Slit skin smear revealed multiple intra- and extracellular leishman donovan bodies with giemsa stain. Biopsy of lesion revealed mixed dermal lymphocytes and histiocytes showing intracellular leishman donovan bodies [Figure 3].

Both the patients were referred to ART center for the management of HIV infection. They were given capsule rifampicin 600 mg od along with ketoconozole 200 mg od for 3 months along with first-line anti-retroviral therapy.

## DISCUSSION

Leishmaniasis is a group of disease caused by several species of genus *leishmania*. Each spices leads to occupy a particular geographical zone, and the disease is endemic in 88 countries. It has been estimated that 1.5 million new cases of cutaneous leishmaniasis occur annually and more than 80% of cases affect individual in developing countries. Brazil, Iran, Afghanistan, and Sudan suffer the highest prevalence and are the hyperendemic regions of the world.[4]

In India, Bihar, Orissa, West Bengal, and northern part of Rajasthan have high prevalence. In old world countries such as India, Bangladesh, Burma, Middle-east and central Asia, Sudan, and Kenya, *leishmania donovani* is a causative organism whereas in new world countries, *Leishmania brazilansis, Leishmania chagasi and Leishmania amazonensis* are the causative organism.[5]

In 5% of East African patients and 20% of Indian patients, a rash develops after visceral disease has been healed, either spontaneously or following treatment.[6] Usually the rash comprises of papules, plaques, and nodules resembling leprosy.[7]

A small proportion of patients with PKDL give no history of visceral disease. In India, rash of PKDL appears 2–3 years after recovery, as hypo pigmented macules.[3] After a variable period of years or months, diffuse nodulation begins to develop in these macules.[8] The rash is progressive and seldom heals spontaneously. Tongue, palate, and genitalia may be involved. There may be lymphadenopathy, but the viscera are spared and there are no features of relapse of the previous systemic infection.

Diagnosis may be clinical, but parasite can be seen by microscopy in smears with limited sensitivity. Polymerase chain reaction (PCR) and monoclonal antibodies may detect parasite in more than 80% cases. Serological test and leishmanin test are of limited value.[3] The aspirates of bone marrow and spleen show extremely heavy parasitization.[9]

Leishmaniasis may be acquired before or after HIV infection. In some patients, typical characteristics of fever and splenomegaly have not been present and serological tests have been negative.[10]

Co-infection of *leishmania* and HIV produces cumulative deficiency of Cell mediated immunity (CMI), a key factor for primary protection against infection, recurrences of metastasis of parasites. Co-infection may amplify the immune defect against both leishmaniasis and HIV and increase disease severity and morbidity. Visceral leishmanaisis, 100-1000 times more common in HIV, is a major fatal outcome of co-infection.[9] PKDL is a complication of visceral leishmaniasis, and it is characterized by macular, maculopapular, and nodular rash in patients who has recovered from visceral leishmaniasis and who is otherwise well. The rash usually starts around mouth, where it spreads to other parts of body depending on severity.

Ramos *et al*. described an HIV-positive patient with visceral leishmaniasis treated with antiretroviral drugs and meglumine antimoniate followed by amphotericin B that after 2 years developed papuloerythematous eruption of post-kala-azar dermal leishmaniasis.[10]

A patient described by Monica Graffanti *et al*. was a HIV-1 infected woman having post-Kala-azar dermal leishmaniasis who recovered after administration of liposomal amphotericin B following failure of oral miltefosine.[11]

Treatment is always needed in Indian patients. Sodium stibogluconate is given at 20 mg/kg for 2 months in Sudan and for 4 months in India. Lyposomal amphotericin B is found to be effective, newer compounds such as miltefosine that can be administered orally or topically.[3]
